# ABHD5 suppresses cancer cell anabolism through lipolysis-dependent activation of the AMPK/mTORC1 pathway

**DOI:** 10.1074/jbc.RA120.014682

**Published:** 2020-11-27

**Authors:** Guohua Chen, Guoli Zhou, Aaron Lotvola, James G. Granneman, Jian Wang

**Affiliations:** 1 Department of Pathology, Wayne State University School of Medicine, Detroit, Michigan, USA; 2 Biomedical Research Informatics Core, Clinical and Translational Sciences Institute, Michigan State University, East Lansing, Michigan, USA; 3 Department of Oncology, Wayne State University School of Medicine, Detroit, Michigan, USA; 4 Center for Molecular Medicine and Genetics, Wayne State University School of Medicine, Detroit, Michigan, USA

**Keywords:** cancer metabolism, lipolysis, αβ hydrolase domain containing 5 (ABHD5), AMP-activated protein kinase (AMPK), mTOR, ABHD5, αβ hydrolase domain containing 5, AMPK, AMP-activated protein kinase, ATGL, adipose triglyceride lipase, ECL, enhanced chemiluminescence, FA, fatty acid, GSEA, gene set enrichment analysis, HSL, hormone sensitive lipase, LDH, lactate dehydrogenase, LD, lipid droplet, mTOR, mechanistic target of rapamycin, OPP, O-propargyl-puromycin, PBS, phosphate-buffered saline, PK, pyruvate kinase, TG, triglyceride

## Abstract

ABHD5 is an essential coactivator of ATGL, the rate-limiting triglyceride (TG) lipase in many cell types. Importantly, ABHD5 also functions as a tumor suppressor, and *ABHD5* mRNA expression levels correlate with patient survival for several cancers. Nevertheless, the mechanisms involved in ABHD5-dependent tumor suppression are not known. We found that overexpression of ABHD5 induces cell cycle arrest at the G1 phase and causes growth retardation in a panel of prostate cancer cells. Transcriptomic profiling and biochemical analysis revealed that genetic or pharmacological activation of lipolysis by ABHD5 potently inhibits mTORC1 signaling, leading to a significant downregulation of protein synthesis. Mechanistically, we found that ABHD5 elevates intracellular AMP content, which activates AMPK, leading to inhibition of mTORC1. Interestingly, ABHD5-dependent suppression of mTORC1 was abrogated by pharmacological inhibition of DGAT1 or DGAT2, isoenzymes that re-esterify fatty acids in a process that consumes ATP. Collectively, this study maps out a novel molecular pathway crucial for limiting cancer cell proliferation, in which ABHD5-mediated lipolysis creates an energy-consuming futile cycle between TG hydrolysis and resynthesis, leading to inhibition of mTORC1 and cancer cell growth arrest.

The proliferation of cancer cells requires a robust synthesis of macromolecules, anabolism, which is essential for the rapid duplication of biomass in the aggressive production of daughter cells. In this regard, proliferating cancer cells usually expand cytosolic glycolysis and repress mitochondrial oxidation so that the carbon skeleton of glucose can be diverted to the anabolic pathways efficiently (1). Despite limited mitochondrial oxidative capacity, robust aerobic glycolysis allows cancer cells to synthesize sufficient ATP to meet the energy needs for proliferation (1). Although the “glucose addiction” of cancer cells is well known, less appreciated is that during rapid cell division, cancer metabolism is often reprogrammed to suppress the utilization of other energy substrates, such as fatty acid (FA), that require mitochondrial oxidation to generate ATP. Such rewiring of metabolic dependency in cancer could represent an unappreciated vulnerability that might be targeted therapeutically.

When cancer cells proliferate under the conditions of high glycolytic flux, FAs are not used as energy substrates and may even be incompatible with fulfilling the energy need for cancer anabolism (2). In this regard, many cancers are known to accumulate excessive amounts of intracellular triglycerides (TG) in lipid droplets (LD) (3, 4, 5, 6, 7, 8, 9, 10), as a result of suppressed lipolysis, the enzymatic hydrolysis of TG. Indeed, tumor progression is accelerated by reduced activity of adipose triglyceride lipase (ATGL, formally PNPLA2), the rate-limiting TG lipase, or loss of αβ hydrolase domain containing 5 (ABHD5), the essential coactivator of ATGL (11, 12, 13, 14, 15, 16, 17). Moreover, we and others have demonstrated that ABHD5, the crucial lipolysis activator, actively participates in tumor suppression (16, 17). While it is clear that activation of the ABHD5/ATGL pathway suppresses cancer cell growth, the mechanisms involved are not fully understood.

The mechanistic target of rapamycin (mTOR) kinase plays a fundamental role in promoting cellular anabolism and is often hyperactive in cancer cells (18). mTOR is associated with two protein complexes, namely mTOR complex 1 (mTORC1) and mTORC2, which have distinct functional outputs in the regulation of cell signaling (18). mTORC1 phosphorylates numerous molecular components that are crucial for activating various biosynthetic pathways. For example, mTORC1 phosphorylates p70S6K kinase at threonine 389 (Thr389) to activate cellular protein synthesis. On the other hand, mTORC2 plays a critical role in finetuning mTOR signaling by phosphorylating a crucial upstream kinase protein kinase B (Akt) (18). Because anabolism is an energy-dependent process, mechanisms exist to restrict mTORC1 activity when energy levels are limiting. Under these conditions, AMP-activated protein kinase (AMPK), the cellular energy sensor, phosphorylates and inhibits mTORC1 activity (19). The metabolites generated by ABHD5/ATGL play a crucial role in the maintenance of energy homeostasis. However, it is presently unclear whether and how lipolysis influences cancer metabolic signaling, including mTOR and AMPK signaling pathways.

In the experiments described below, we characterized the effects of genetic and pharmacologic activation of ABHD5 on cancer cell growth. We report that ABHD5-induced growth arrest is mediated by ATGL and is associated with inhibition of mTORC1-mediated protein synthesis. Mechanistically, lipolysis activation leads to the ATP-consuming process of FA re-esterification, which elevates AMP and activates AMPK. These results elucidate a critical connection between the metabolic and signaling pathways that support cancer growth and further reveal the vulnerability of cancer cells to activation of the ABHD5/ATGL metabolic pathway. Since ABHD5 can be targeted by small ligands (20, 21), these results pave an avenue toward developing new cancer therapeutics through pharmacological activation of ABHD5-mediated lipolysis.

## Results

### Overexpression of ABHD5 induces cell cycle arrest and inhibits cell proliferation

**Figure 1:**
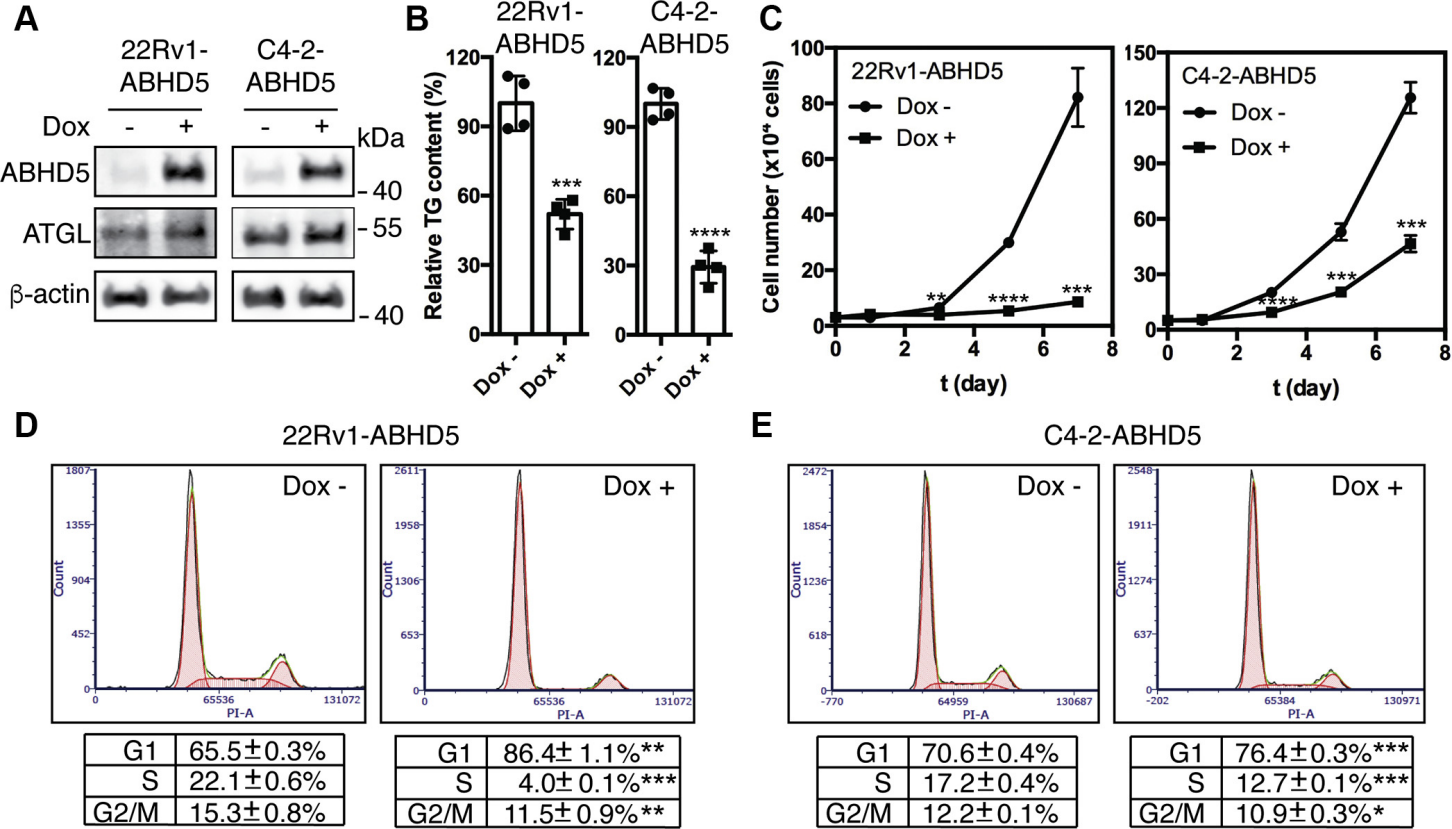
**Overexpression of ABHD5 induces cell cycle arrest and inhibits the proliferation of cancer cells**. *A*, western blotting for the level of ABHD5, ATGL, and β-actin proteins in the 22Rv1-ABHD5 and C4-2-ABHD5 transgenic cells with or without doxycycline treatment for 48 h. *B*, relative intracellular triacylglycerol (TG) levels in the 22Rv1-ABHD5 and C4-2-ABHD5 transgenic cells with or without doxycycline treatment for 72 h. Data are presented as mean ± SD (n = 4). ∗∗∗*p* < 0.001, ∗∗∗∗*p* < 0.0001. *C*, cell growth kinetics of the 22Rv1-ABHD5 (*left*) and C4-2-ABHD5 (*right*) cells in the absence (*circle*) or presence (*square*) of doxycycline for up to 7 days. Data are presented as mean ± SD (n = 3). ∗∗*p* < 0.01, ∗∗∗*p* < 0.001, ∗∗∗∗*p* < 0.0001. *D*–*E*, flow cytometry to profile the cell cycle distribution of the 22Rv1-ABHD5 (*D*) and C4-2-ABHD5 (*E*) cells grown in the absence (*left*) and presence (*right*) of doxycycline for 72 h. Data are presented as mean ± SD (n = 3). ∗*p* < 0.05, ∗∗*p* < 0.01, ∗∗∗*p* < 0.001, ∗∗∗∗*p* < 0.0001. Student *t*-test.

To test the effects of ABHD5 on cancer cell growth, we engineered stable ABHD5 overexpression in two castration-resistant prostate cancer cell lines, 22Rv1 (22) and C4-2 cells (23), under the control of a doxycycline (Dox)-inducible promoter. These transgenic cell models are designated as 22Rv1-ABHD5 and C4-2-ABHD5 cells, respectively. Dox treatment of the 22Rv1-ABHD5 and C4-2-ABHD5 cells significantly elevated the cellular levels of ABHD5 protein and slightly increased the levels of ATGL (Fig. 1*A*). As expected, ABHD5 induction significantly reduced the cellular TG contents of both cell lines (Fig. 1*B*). Next, we determined the ABHD5 effects on cell growth kinetics by measuring the cell counts of 22Rv1-ABHD5 and C4-2-ABHD5 cells growing in the absence or presence of Dox over time. Dox treatment had a negligible effect on the growth of the 22Rv1 and C4-2 parent cell lines (Data not shown). Interestingly, Dox-induced ABHD5 expression inhibited proliferation of both cell lines over a 7-day time course (Fig. 1*C*). We also confirmed the potent inhibition of cell proliferation by ABHD5 overexpression in an additional prostate cancer cell line LNCaP cells (Fig. S1). To gain the mechanistic insight into how ABHD5 suppresses cell proliferation, we compared the cell cycle distributions of the cells using flow cytometry. Overexpression of ABHD5 for 72 h significantly increased the G1 phase cell population from 65.5% to 86.4% for 22Rv1-ABHD5 (*p* < 0.01) and from 70.6% to 76.6 for C4-2-ABHD5 (*p* < 0.001) and decreased the S phase cell population from 22.1% to 4.0% for 22Rv1-ABHD5 (*p* < 0.001) and from 17.2% to 12.7% for C4-2-ABHD5 (*p* < 0.001) (Fig. 1, *D* and *E*). ABHD5 induction modestly increased cellular levels of p53 (Fig. S2). However, ABHD5 overexpression did not enhance PARP-1 cleavage, indicating its negligible effect to induce apoptosis (Fig. S2). These results indicate that ABHD5 has a strong capacity for inducing G1 cell cycle arrest in prostate cancer cells, possibly resulting in part from activation of the tumor suppressor p53.

### Activation of lipolysis impedes mTORC1 signaling and inhibits cellular protein biosynthesis

**Figure 2:**
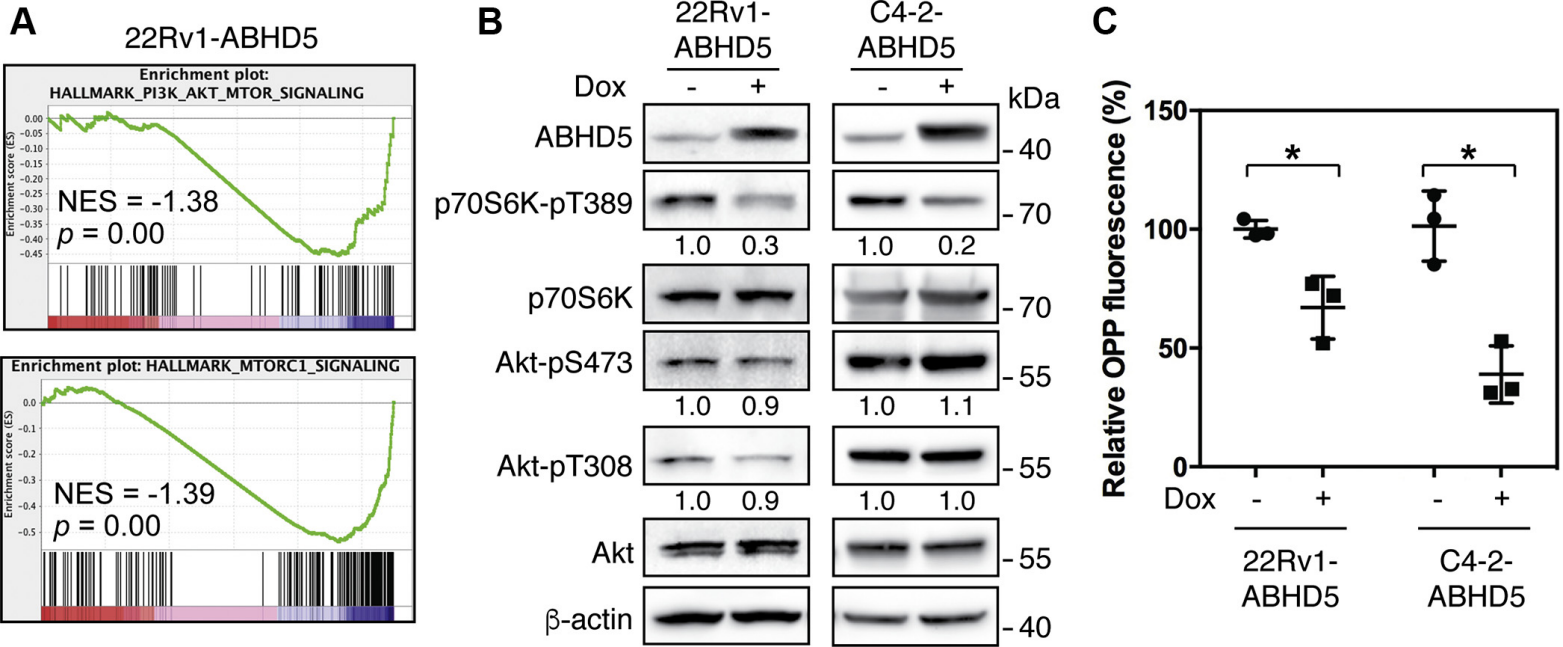
**ABHD5 suppresses mTORC1 signaling and protein synthesis**. *A*, gene set enrichment plots to demonstrate significant downregulation of PI3K/Akt/mTOR (*top*) and mTORC1 (*bottom*) signaling pathways by ABHD5 overexpression in 22Rv1 cells, identified by QuantSeq gene expression profiling followed by GSEA analysis. *B*, western blotting for the level of the indicated proteins in the 22Rv1-ABHD5 (*left*) and C4-2-ABHD5 (*right*) cells with or without doxycycline treatment for 24 h. Relative band intensities are shown at the bottom of the blots. *C*, relative OPP fluorescence intensity to label the level of new protein synthesis in the 22Rv1-ABHD5 and C4-2-ABHD5 cells with or without doxycycline treatment for 48 h. Data are presented as mean ± SD (n = 3). ∗*p* < 0.05. Student *t*-test.

To investigate the molecular basis by which ABHD5 inhibits the proliferation of cancer cells, we profiled the transcriptomes of 22Rv1-ABHD5 cells grown in the presence and absence of Dox. Using gene set enrichment analysis (GSEA) (24), we then identified the critical molecular pathways that are regulated by ABHD5 expression. We found that ABHD5 expression strongly repressed the PI3K/Akt/mTOR signaling (NES = –1.38, *p* = 0) and mTORC1 signaling (NES = –1.39, *p* = 0) pathways (Fig. 2*A*). To assess the activity of these pathways, we measured the phosphorylation status of Akt and p70S6K at the two key signaling nodes. In both 22Rv1-ABHD5 and C4-2-ABHD5 cells, we found that ABHD5 induction significantly decreased the phosphorylation of p70S6K at Thr389, a molecular surrogate measuring the mTORC1 activity, without influencing either Thr308 or Ser437 phosphorylation of Akt that measures the strength of PI3K activity and the mTORC2 activity, respectively (Fig. 2*B*) (18). Thus, the induction of ABHD5 selectively suppresses the mTORC1 activity on the PI3K/Akt/mTOR pathway.

A canonical functional output of the mTORC1 signaling lies in its activation of anabolic pathways, including activation of protein synthesis. We then used the O-propargyl-puromycin (OPP) fluorescence probe (25) to quantify cellular synthesis of nascent peptides. We found that ABHD5 induction by Dox treatment significantly decreased the fluorescence labeling intensity (Fig. 2*C*). Thus, these results indicate that the elevation of ABHD5 expression suppresses mTORC1 signaling and inhibits cellular protein synthesis.

**Figure 3:**
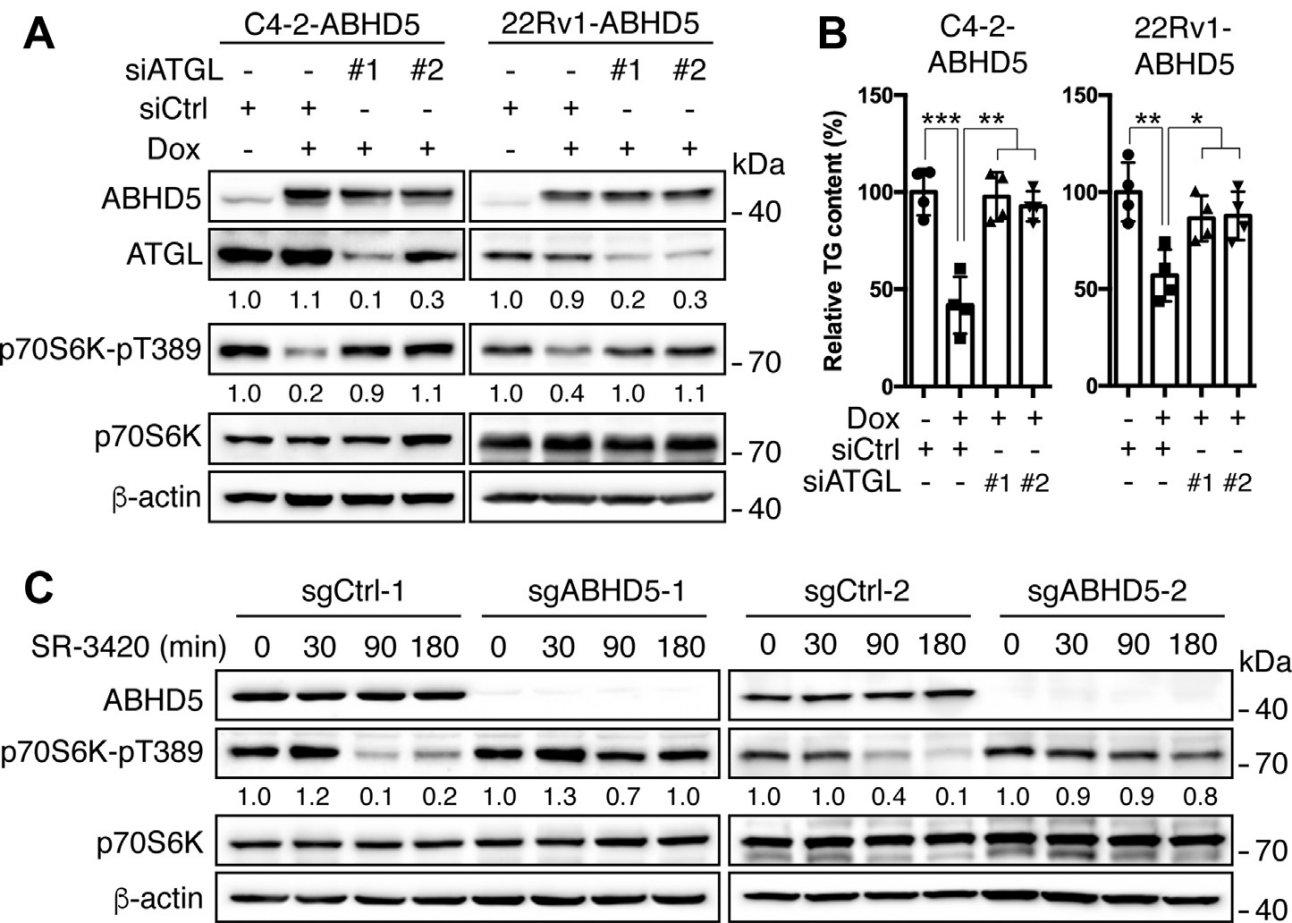
**ABHD5-mediated mTORC1 suppression involves lipolytic activity**. *A*, western blotting to demonstrate the level of the indicated proteins in the C4-2-ABHD5 (*left*) and 22Rv1-ABHD5 (*right*) cells with or without doxycycline treatment and ATGL siRNA knockdown. Relative band intensities are shown at the bottom of the blots. *B*, relative intracellular triacylglycerol (TG) levels in the cells treated as in *A*. Data are presented as mean ± S.D. (n = 4). ∗*p* < 0.05, ∗∗*p* < 0.01, ∗∗∗*p* < 0.001. Student *t*-test. *C*, western blotting to demonstrate the effects of SR-3420 (10 μM) on the expression of the indicating proteins in the wild-type (sgCtrl) and ABHD5-deficient (sgABHD5) C4-2 cells for the indicated time. Relative band intensities are shown at the bottom of the blots.

ABHD5 is well known to coactivate the ATGL TG lipase and thus stimulate lipolysis (26). We then knocked down ATGL to investigate whether ATGL-mediated lipolytic activity is critically involved in the mTORC1 suppression by ABHD5. The results showed that transfection of two independent small interfering RNAs for ATGL each decreased the level of ATGL protein by >70% with negligible influence on the level of ABHD5 protein in both C4-2-ABHD5 and 22Rv1-ABHD5 cells (Fig. 3*A*). As expected, knockdown of ATGL blocked the downregulation of cellular TG contents by ABHD5 overexpression (Fig. 3*B*). Importantly, knockdown of ATGL significantly blunted the downregulation of p70S6K Thr389 phosphorylation by ABHD5 induction (Fig. 3*A*), indicating a dependence on ATGL by ABHD5 to inhibit mTORC1.

SR-3420 is a highly selective synthetic ligand of ABHD5 that potently activates ATGL-dependent lipolysis (20, 21). Treatment of C4-2 cells with SR-3420 strongly suppressed p70S6K Thr389 phosphorylation in a time-dependent manner (Fig. 3*C*). To further verify the specificity of SR-3420 against ABHD5, we generated clones of C4-2 cells with targeted deletion of *ABHD5* using the CRISPR-Cas9 system. Using this model, we observed that the knockout of ABHD5 reduced the downregulation of p70S6K Thr389 phosphorylation by SR-3420 treatment in the two ABHD5-deficient cell clones generated with independent sgRNAs (Fig. 3*C*). Collectively, activation of ABHD5-mediated lipolysis, genetically or pharmacologically, impedes mTORC1 signaling and inhibits the anabolism of cancer cells.

### Activation of lipolysis triggers triglyceride hydrolysis–synthesis futile cycle, resulting in AMPK activation and thus mTORC1 inhibition

**Figure 4:**
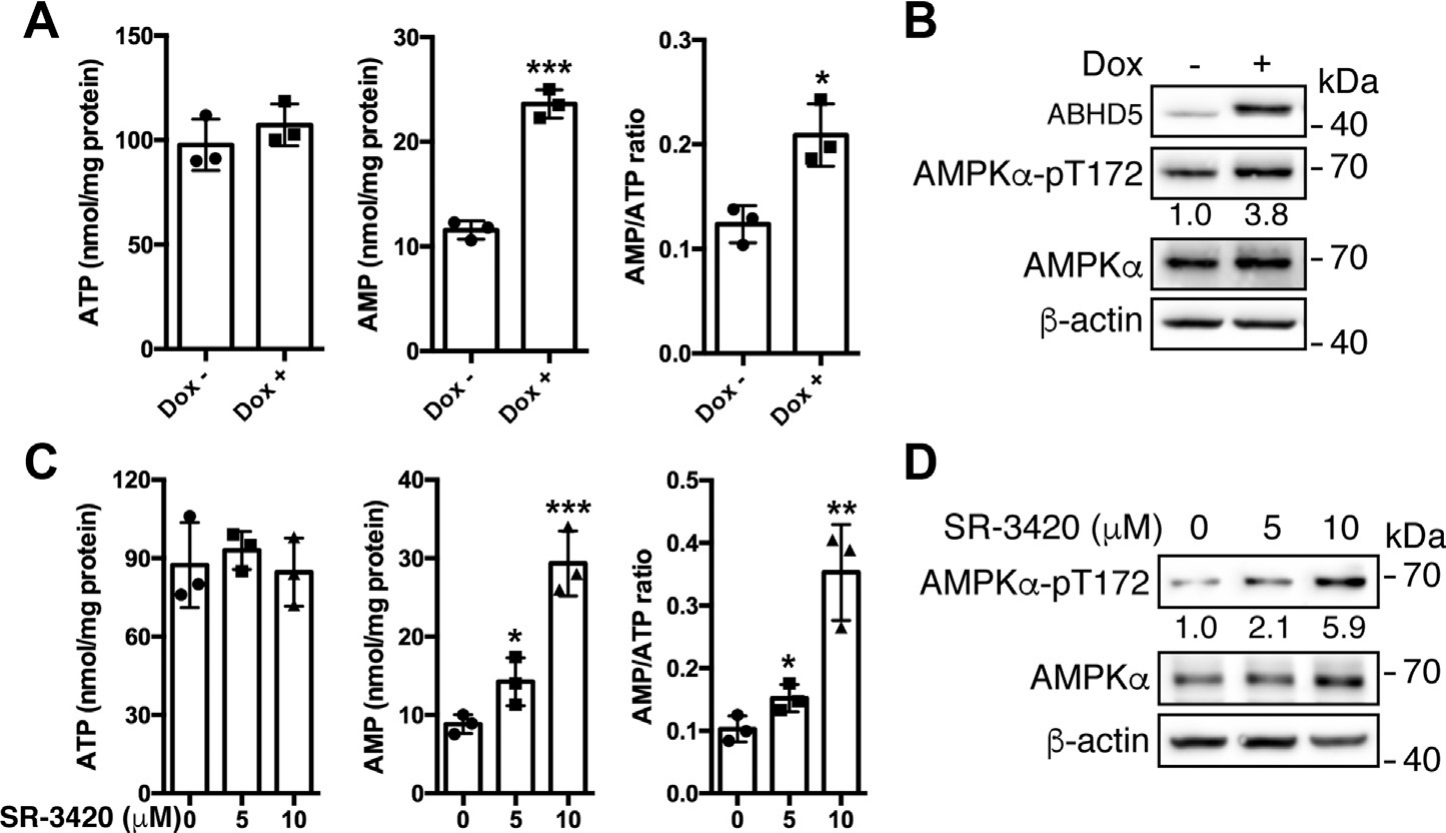
**Overexpression of ABHD5 induces energy stress and activates AMPK**. *A*, bar graphs to illustrate the level of AMP, ATP, and AMP/ATP ratio in the C4-2-ABHD5 cells with or without doxycycline treatment for 24 h. *B*, western blotting to demonstrate the level of the indicated proteins in C4-2-ABHD5 cells as treated in *A*. Relative band intensities are shown at the bottom of the blots. *C*, bar graphs to illustrate the level of AMP, ATP, and AMP/ATP ratio in the C4-2 cells treated with the indicated amount of SR-3420 for 90 min. *D*, western blotting to demonstrate the level of the indicated proteins in the C4-2 cells treated as in *C*. Relative band intensities are shown at the bottom of the blots. ∗*p* < 0.05, ∗∗*p* < 0.01, ∗∗∗*p* < 0.001. Student *t*-test. Data are presented as mean ± S.D. (n = 3).

Lipolysis is functionally associated with the regulation of energy homeostasis (26). We thus measured the intracellular ATP and AMP abundance in the C4-2-ABHD5 cells with or without Dox-inducible ABHD5 expression. The results showed that while the ATP level remained flat in the ABHD5-overexpressing cells, the AMP level significantly increased, leading to a significant elevation of AMP/ATP ratio (Fig. 4*A*). As expected, overexpression of ABHD5 activated AMPKα as indicated by its elevated phosphorylation at Thr172 (Fig. 4*B*). Next, we investigated the effects of SR-3420 on the energy status of the cells. The results showed that the treatment of C4-2 cells with SR-3420 elicited a dose-dependent increase of intracellular AMP level and AMP/ATP ratio (Fig. 4*C*). Also, SR-3420 increased the levels of AMPKα Thr172 phosphorylation in parallel with the elevation of AMP/ATP ratios (Fig. 4*D*). Thus, genetic or pharmacologic activation of ABHD5 induces energy stress and activates AMPK.

**Figure 5:**
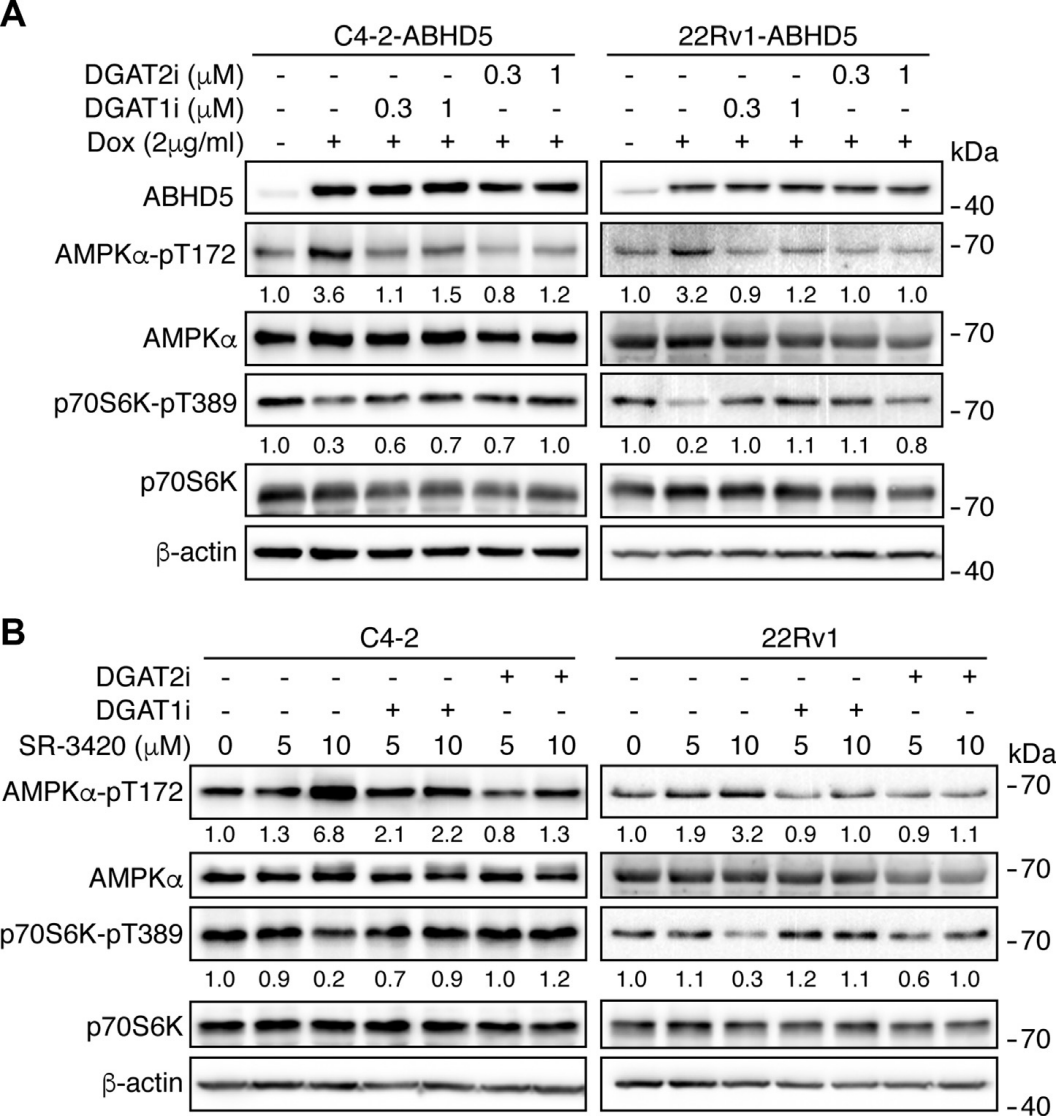
**Activation of AMPK by lipolysis involves DGAT activity**. *A*, western blotting to analyze the level of the indicated proteins in the C4-2-ABHD5 (*left*) and 22Rv1 (*right*) cells with or without doxycycline, PF04620110 (DGAT1i, 0.3 or 1 μM), and PF06424439 (DGAT2i, 0.3 or 1 μM) treatment for 24 h. *B*, western blotting to analyze the level of the indicated proteins in the C4-2 (*left*) and 22Rv1 (*right*) cells with the indicated amount of SR-3420 treatment alone or in combination with 10 μM PF04620110 (DGAT1i) or PF06424439 (DGAT2i) treatment for 90 min. Relative band intensities are shown at the bottom of the blots.

The FAs derived from lipolysis may feed mitochondrial oxidation, which promotes ATP synthesis and thus enhances cellular energy level. In contrast, the mobilized FAs can be re-esterified into TG in a process consuming ATP, thus reducing cellular energy level (27). We thus used chemical inhibitors to probe how these metabolic pathways would link lipolysis activity to the modulation of cellular energy state and signaling. Inhibition of mitochondrial FA oxidation with etomoxir (28) had negligible effects on the cellular AMP, ATP contents, and AMPK activation by ABHD5 overexpression in C4-2-ABHD5 cells (Fig. S3), suggesting that lipid oxidation may be a minor source to support energy production in these cells. However, inhibition of FA re-esterification by DGAT1 or DGAT2 inhibitors PF04620110 (29) or PF06424439 (30) blunted the activation of AMPK by ABHD5 induction (Fig. 5*A*) or SR-3420 treatment (Fig. 5*B*) in both C4-2 and 22Rv1 cells. These results indicate that lipolysis-triggered AMPK activation involves FA re-esterification and strongly suggest that a futile cycle involving active hydrolysis and resynthesis of TG dissipates cellular energy and activates AMPK signaling in response to lipolysis activation in these cancer cells.

**Figure 6:**
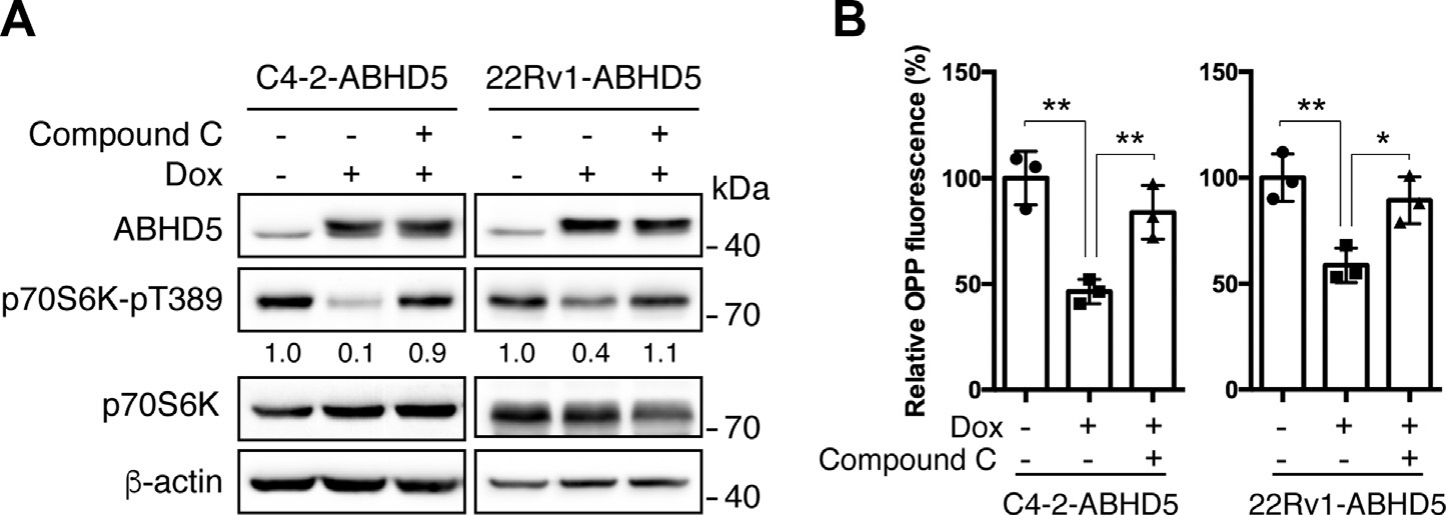
**AMPK mediates the mTORC1 suppression by ABHD5**. *A*, western blotting to analyze the level of the indicated proteins in C4-2-ABHD5 (*left*) and 22Rv1-ABHD5 (*right*) cells with or without doxycycline and 1 μM Compound C treatment for 24 h. Relative band intensities are shown at the bottom of the blots. *B*, OPP fluorescence labeling to analyze the new protein synthesis in C4-2-ABHD5 (*left*) and 22Rv1-ABHD5 (*right*) cells treated as in *A*. Data are presented as mean ± SD (n = 3). ∗*p* < 0.05, ∗∗*p* < 0.01. Student *t*-test.

AMPK strongly inhibits cellular anabolism through the inactivation of mTORC1 signaling (19). To test the possibility that AMPK mediates ABHD5-dependent inhibition of mTORC1, we used Compound C (31) to inhibit AMPK in C4-2-ABHD5 and 22Rv1-ABHD5 cells. We observed that the treatment of Compound C significantly attenuated the suppression of ABHD5 on p70S6K Thr389 phosphorylation (Fig. 6*A*) and new protein synthesis, as shown by OPP labeling (Fig. 6*B*). These results indicate that AMPK activation links lipolysis activity to the suppression of mTORC1 signaling and anabolic metabolism in these cancer cells.

Collectively, these experiments identified a novel regulatory cascade through which activation of ABHD5 inhibits cancer anabolism by creating a futile cycle of TG hydrolysis and synthesis that activates AMPK and subsequently inhibits mTORC1.

### *ABHD5* confers a better prognosis for multiple cancer types

**Figure 7:**
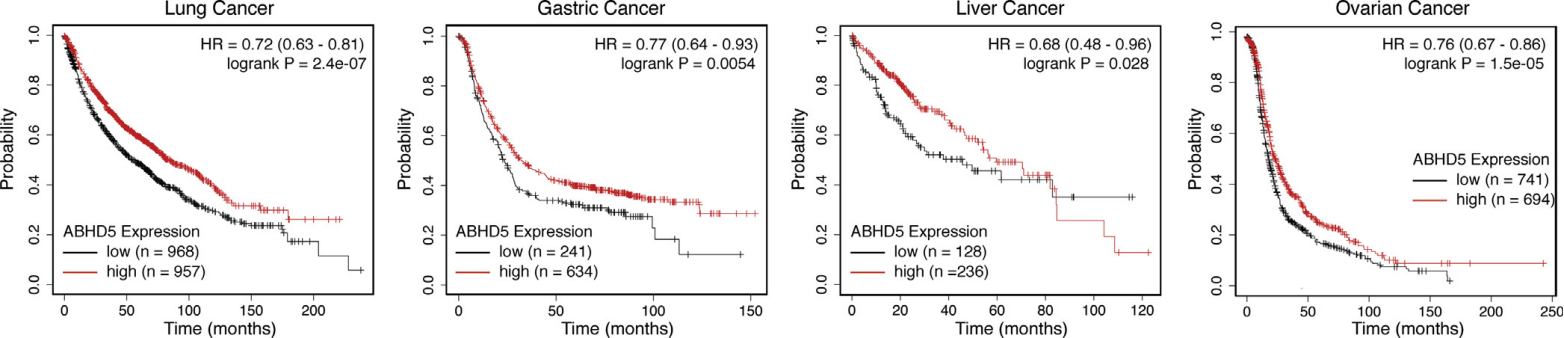
**High *ABHD5* expression is associated with better patient survival in multiple cancer types**. Kaplan–Meier survival analyses reveal the prognostic values of the *ABHD5* gene expression in the lung, gastric, liver, and ovarian cancers.

The above results strongly suggest a therapeutic potential of activation of ABHD5-mediated lipolysis in the treatment of cancer diseases. To address this possibility, we examined whether *ABHD5* gene expression levels correlated with cancer patient survival data in the publicly accessible database, the Kaplan–Meier Plotter (32). Interestingly, among the total of five cancer types available in the database, we found that high *ABHD5* expression significantly correlates with extended patient survival in four cancer types, including lung cancer (HR = 0.72, log-rank *p* = 2.4e-07), gastric cancer (HR = 0.77, log-rank *p* = 0.0054), liver cancer (HR = 0.68, log-rank *p* = 0,028), and ovarian cancer (HR = 0.76, log-rank *p* = 1.5e-05) (Fig. 7). By overexpressing ABHD5 in the lung cancer cell line H1299, we observed that ABHD5 significantly reduces the ability of these cells to synthesize nascent proteins (Fig. S4), which provides initial evidence that ABHD5 suppresses the cellular anabolism of another cancer type in addition to the prostate cancer cells. Collectively, these results indicate that ABHD5 protects against patient death in multiple cancer types and that activation of ABHD5 may represent a promising therapeutic opportunity against these cancers.

## Discussion

We and others previously demonstrated that loss of ABHD5 promotes aerobic glycolysis and stimulates migration and invasion of prostate and colorectal cancer cells (16, 17). Surprisingly, knockdown of ATGL, the primary target of ABHD5, inhibited cell proliferation and migration (16, 17), raising a critical question of whether ABHD5 and ATGL can functionally interact to regulate cancer cell metabolism. Although ABHD5 is an essential activator for TG lipase ATGL, it was unclear whether the cancer suppression by ABHD5 involves lipolytic activity and, if so, the signaling pathways involved. Here, we demonstrate that ABHD5 inactivates mTORC1 signaling in an ATGL-dependent manner, resulting in suppression of protein synthesis and G1 cell cycle arrest. Previous studies demonstrated that inactivation of ATGL or hormone sensitive lipase (HSL) in mice significantly sensitizes insulin signaling and improves glucose tolerance in multiple metabolic organs (33, 34). Strikingly, double adipose deletion of *ATGL* and *HSL* in mice resulted in liposarcoma with a full penetration (35). It is likely that lipolysis can act as a functional barrier against anabolic pathways at both physiological and pathological levels. Collectively, our results not only revealed the mechanisms underlying the inhibition of cell proliferation by ABHD5 but also underscore the possibility of targeting cancer by activation of lipolysis.

Tumor tissues are more often enriched with LD as compared with normal tissues. In addition to the well-known cytotoxicity associated with excessive FAs, the current study revealed a new functional role of the lipolytic product in the suppression of cancer cell anabolism. Therefore, holding a significant reservoir of FAs in the form of intracellular TG is potentially an “Achilles heel” of cancer cells that are susceptible to lipolysis activation. Using SR-3420, a highly selective synthetic agonist for ABHD5-mediated lipolysis (20, 21), we demonstrated that pharmacological activation of ABHD5 potently inhibits mTORC1, the master signaling driver for cancer cell anabolism. These results provide a proof of the concept for potential cancer intervention by pharmaceutical targeting of ABHD5. In this regard, mining the data of cancer patient survival revealed that high ABHD5 expression is associated with significantly better prognosis in four different cancer types, suggesting that ABHD5 activation might be developed into an effective therapy in a broad spectrum of cancer diseases.

How ABHD5-mediated lipolysis inactivates mTORC1 signaling is an intriguing mechanistic question. Because lipolysis releases FAs as efficient energy substrates, an underlying possibility would be that ABHD5-mediated lipolysis alters cellular energy state and triggers metabolic signaling. Indeed, we observed that ABHD5 activation induces cellular AMP accumulation and AMPK activation that is required for mTORC1 suppression. We thus identified AMPK as a crucial signal transducer connecting the cross talk between ABHD5 and mTORC1. Interestingly, these results further suggest a novel cell-autonomous mechanism by which activation of lipolysis signals energy stress to the restriction of anabolic activities in cancer cells.

During active lipolysis in adipocytes, a significant fraction of mobilized FA is re-esterified to TG by DGAT enzymes (27). Because FA esterification requires ATP-consuming acyl-CoA synthesis, active cycling between TG hydrolysis and resynthesis dissipates energy and prevents ER stress (36, 37). Similarly, we demonstrated that the AMPK activation by ABHD5 is sensitive to the inhibition of DGAT enzymes. Therefore, our results suggest that an energy-consuming futile cycle of FA generation and esterification is responsible for AMP accumulation and AMPK activation downstream of ABHD5 activation in cancer cells. However, FAs derived from lipolysis can fuel mitochondrial oxidation and promote ATP generation. It is presently unclear how the distribution of FA flux between TG synthesis and mitochondrial oxidation is regulated. Cancer cells exhibiting Warburg metabolism have reduced mitochondrial oxidative capacity, which may favor a diversion of FA flux to the TG synthesis pathway, resulting in ATP dissipation. In this regard, our findings underlie the significance for further characterization of the metabolic checkpoint that controls the dynamics of FA fluxes in cancer cells.

## Experimental procedures

### Cell culture, plasmid construct, viral packaging, and cell line generation

Human prostate cancer cell lines LNCaP, 22Rv1 and C4-2 (ATCC) were maintained in RPMI1640 medium supplemented with 10% fetal bovine serum (FBS) and 100 U of penicillin/ml, and 0.1 mg of streptomycin/ml. HEK293FT (Invitrogen) cells and human lung cancer cell line H1299 (ATCC) were maintained in DMEM medium supplemented with 10% FBS and 100 U of penicillin/ml, and 0.1 mg of streptomycin/ml. For pharmacological manipulation of the specific enzymes, SR-3420 was described previously (21), and PF04620110, PF06424439, and Compound C were purchased from Tocris Bioscience.

For doxycycline-inducible overexpression of ABHD5, the pInducer20-ABHD5 lentiviral vector was constructed as described previously (17). For constitutive overexpression of ABHD5, the cDNA encoding the full-length human ABHD5 was fused with an in-frame C-terminal Flag tag and cloned into the Nhe1/EcoR1 sites of pcDNA_3.1_.puro, a pcDNA_3.1_ derivative with the replacement of the neomycin-resistant with a puromycin-resistant cassette. For construction of the targeting vector against human ABHD5, the sgRNA sequences were determined using a web bioinformatics tool (http://crispr.mit.edu) (38). The oligo DNAs that encode sgRNA were annealed and cloned into pSpCas9(BB)-2A-Bsd, a modified vector from pSpCas9(BB)-2A-Puro (PX459, Addgene) made by replacing the puromycin-resistant to a blasticidin-resistant gene. The sequences of sgRNA for ABHD5 are 5ʹ-TTAAGGTGTGATATAGACGT-3ʹ (sgABHD5-1) and 5ʹ-TTGGACGAAGTAGTAGACCC-3ʹ (sgABHD5-2).

For lentiviral packaging, pInducer20-ABHD5 vector was cotransfected with pMD2.G and psPAX2 packaging vectors into HEK293FT cells using lipofectamine 2000 (Invitrogen). 48 h posttransfection, virus-containing culture media were harvested and cell debris were removed by passing through 0.45 μm filters.

For inducible overexpression of ABHD5, LNCaP, 22Rv1, and C4-2 cells were infected with pInducer20-ABHD5 lentivirus and selected with 500 μg G418/ml. For constitutive overexpression of ABHD5, H1229 cells were transfected with pcDNA_3.1_.puro-ABHD5 and selected with 2 μg/ml puromycin. For knockout of ABHD5, C4-2 cells were transfected with pSpCas9(BB)-2A-Bsd-ABHD5 targeting vector and selected with 5 μg blasticidin/ml. The drug-resistant cells were validated for altered ABHD5 expression by western blotting.

### Western blotting

Cells were washed twice in phosphate-buffered saline (PBS) and lysed in cytoplasmic lysis buffer (25 mM Tris-HCl pH 7.5, 40 mM NaCl, 50 mM NaF, 2 mM Na_2_VO_4_, 1% Triton X-100). Protein concentrations were determined with the BCA reagent (Pierce). Cell lysates (40 μg) were resolved by sodium dodecyl sulfate–polyacrylamide gel electrophoresis (SDS-PAGE), and proteins were transferred onto nitrocellulose filters. The blots were saturated with 5% BSA and probed with antibodies against ABHD5 (#12201-1-AP, Proteintech, 1:1000), ATGL (#2138, Cell Signaling, 1:1000), phospho-Akt (Ser473) (#4060, Cell Signaling, 1:1000), phospho-Akt (Thr308) (#4056, Cell Signaling, 1:1000), Akt (#2920, Cell Signaling, 1:1000), phosphor-p70S6K (#9205, Cell Signaling, 1:1000), p70S6K (#2708, Cell Signaling, 1:1000), phosphor-AMPKα (Thr172) (#2535, Cell Signaling, 1:1000), AMPKα (#5831, Cell Signaling, 1:1000), PARP1 (#13371-1-AP, Proteintech, 1:1000), p53 (#MA5-12571, Invitrogen, 1:1000), β-actin (A2066, Sigma, 1:1000). Following a wash with TBST (TBS containing 0.1% Tween 20), the blots were incubated with peroxidase-coupled goat anti-rabbit immunoglobulin G (Sigma, 1:5000). The immunolabeled protein bands were detected by enhanced chemiluminescence (ECL) method (Perkin Elmer). The blot images were acquired on an Azure 600 imager (Azure biosystems), and the protein band intensities were quantified using the ImageJ software.

### Determination of cell proliferation and cell cycle profile

Cells were seeded on 6-cm plates and grown for the indicated time intervals in the presence or absence of 2 μg/ml doxycycline. The dead cells were excluded with trypan blue staining (Invitrogen), and the number of live cells was measured using with a hemocytometer under a light microscope.

For the measurement of cellular DNA content, cells were fixed in 70% ethanol and stained with 5 μg/ml propidium iodide and sorted using flow cytometry.

### Measurement of intracellular triacylglycerol

To measure intracellular triacylglycerol, cells were harvested and suspended in 5% NP-40 aqueous solution. Cell extracts were prepared with two cycles of heating (95 °C, 5 min) and cooling (on ice, 5 min) followed by a centrifugation at 12,000*g* for 5 min. Cellular triacylglycerol contents were determined with TG reagent (#T2449, Sigma) on a microplate reader (BMG Labtech) according to the manufacturer’s specifications.

### RNA-seq transcriptomics profiling and GSEA analysis

Total cellular RNA was isolated using the TRIzol reagent (Invitrogen), and cDNA libraries compatible for Illumina sequencing were prepared by using the QuantSeq 3ʹ mRNA-seq Reverse (REV) Library Prep Kit (Lexogen) according to the manufacturer’s instruction. The resultant cDNA libraries were assessed using a TapeStation (Agilent) and subjected to 100 bp single end sequencing using the Illumina HiSeq 2500 system at the Wayne State University Applied Genomics Technology Center. Raw sequencing reads (FASTQ) were demultiplexed using Illumina’s CASAVA 1.8.2 software and processed with FastQC to remove low-quality and unknown sequences. To quantify transcript abundance, the processed reads were mapped to the hg38 human reference genome using STAR, and the differential gene expression was determined using edgeR. The RNA-Seq data were uploaded to GEO database with the accession GSE151957. The pathway enrichment analysis was performed using the web-based GSEA software (http://software.broadinstitute.org/gsea) with the following parameters: 1000 permutations, mode run on preranked gene list from the MSigDB hallmark gene sets, enrichment statistic “classic.”

### Metabolite extraction and measurements of AMP and ATP

Cells were quickly rinsed with ice-cold PBS for two times and then snap-frozen in 50% aqueous methanol on dishes in liquid nitrogen. Following three cycles of freezing in liquid nitrogen and thaw at 37 °C, cell debris was clarified by centrifugation at 10,000*g* at 4 °C for 10 min. The hydrophilic metabolites were dried on a lyophilizer and reconstituted in H_2_O before the metabolite measurement.

ATP was measured using the commercial ATP Determination Kit (#A22066, Molecular Probes) per vendor’s specification. AMP was measured using a method adapted from a previous report (39). Briefly, AMP was quantified by recording the fluorescence intensity (ex = 340 nm, em = 450 nm) in a reaction mix containing 20 mM imidazole-HCl, 75 mM KCl, 2 mM MgCl_2_, 5 μM ATP, 20 μM phosphoenolpyruvate, 10 μM NADH, 0.4 U/ml lactate dehydrogenase (LDH), 0.3 U/ml pyruvate kinase (PK), and 0.4 U/ml myokinase on a microplate reader. LDH, PK, and myokinase were added to the reaction sequentially, and the endpoint fluorescence intensities were measured at 5 min after the addition of each enzyme. Cellular AMP content was extrapolated using the decrease of the fluorescence catalyzed by myokinase in proportion to the reference standards.

### Measurement of protein synthesis

The labeling and quantitation of cellular nascent protein production were performed using the commercial Click-it Plus OPP Alexa Fluor 488 Protein Synthesis Assay Kit (#C10456, Molecular Probes) on a microplate reader per vendor’s specification.

### Cancer patient survival analysis

The influence of ABHD5 expression in cancer patient survival was analyzed using the web-based database (http://kmplot.com) with the following parameters: auto select best cutoff, survival: OS. The Affy id for the ABHD5 mRNA is 213935_at in the analyses for lung, gastric, and ovarian cancer. The Rna-seq id for the ABHD5 mRNA is 51099 in the analysis for liver cancer.

## Data availability

All data are contained within the article.

## Conflict of interest

The authors declare that they have no conflicts of interest with the contents of this article.
