# Supplementary material for: ABHD5 suppresses cancer cell anabolism through lipolysis-dependent activation of the AMPK/mTORC1 pathway
Source: J Biol Chem. 2020 Nov 27;296:100104. doi: 10.1074/jbc.RA120.014682 (PMC7949079; doi:10.1074/jbc.RA120.014682)
Supplement: Supplementary file 1 — Figures S1 to S4 [file mmc1.pdf]

Supporting Information

ABHD5 suppresses cancer cell anabolism through lipolysis-dependent activation of the AMPK/mTORC1 pathway

Guohua Chen[1], Guoli Zhou[2], Aaron Lotvola[3], James G. Granneman[4] and Jian Wang[1*]

[1]Department of Pathology, [3]Department of Oncology, and [4]Center for Molecular Medicine and Genetics, Wayne State University School of Medicine, Detroit, MI 48202, USA

[2] Biomedical Research Informatics Core, Clinical and Translational Sciences Institute, Michigan State University, East Lansing, MI 48824 USA

*Corresponding author: Jian Wang
Email: jianwang@med.wayne.edu

Running title: Lipolysis tunes down cancer anabolism

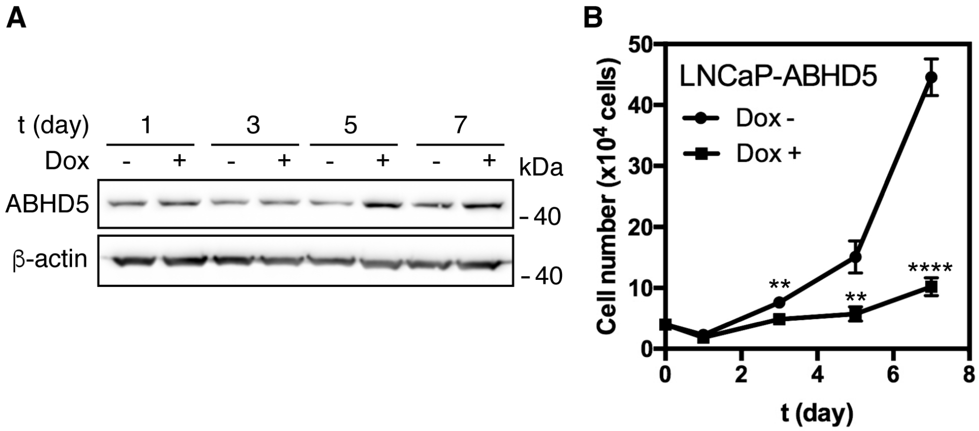

Figure S1. Overexpression of ABHD5 inhibits the proliferation of LNCaP cells.

A. Western blotting for the levels of ABHD5 and β-actin in the LNCaP-ABHD5 cells with or without doxycycline treatment for the indicated time.

B. Cell growth kinetics of the LNCaP-ABHD5 cells in the absence (circle) or presence (square) of doxycycline for up to 7 d. Data are presented as Mean ± SD (n = 3). **$p<0.01$, ****$p<0.0001$.

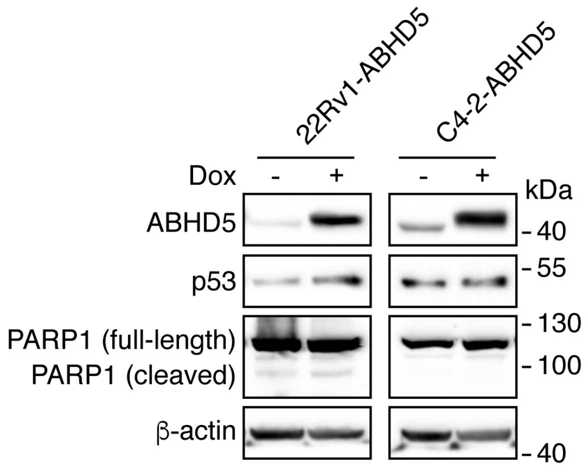

Figure S2. Overexpression of ABHD5 modestly stimulates p53 expression but does not induce apoptosis.

Western blotting to determine the levels of the indicated proteins in the 22Rv1-ABH5 (left) and C4-2-ABHD5 (right) cells with or without doxycycline (Dox) treatment or 72 h.

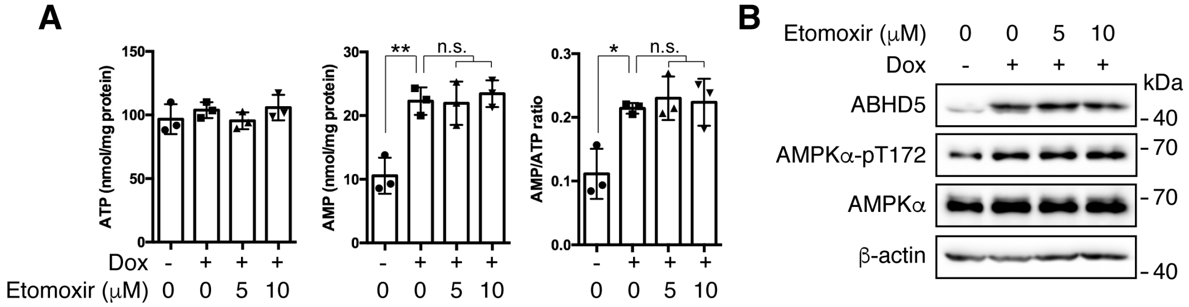

Figure S3. Etomoxir does not prevent ABHD5 from activating AMPK in C4-2 cells.

A. Levels of AMP, ATP, and AMP/ATP ratio in the C4-2-ABHD5 cells treated as indicated for 24 h. Data are presented as Mean ± SD (n = 3). *p<0.05, **p<0.01, n.s. not significant. Student t-test.

B. Levels of the indicated proteins in the C4-2-ABHD5 cells treated as in A.

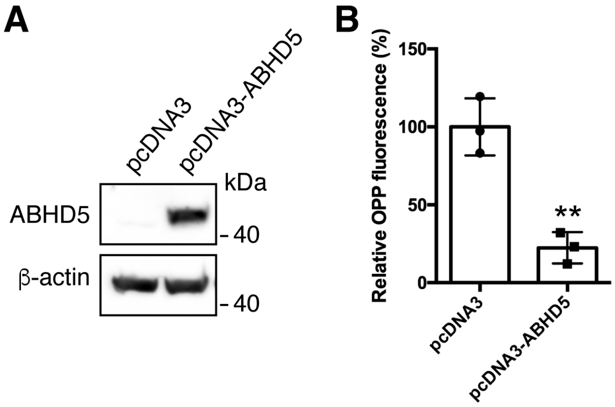

Figure S4. Overexpression of ABHD5 inhibits the protein synthesis of human lung cancer H1299 cells.
Levels of the indicated proteins (A) and relative new protein synthesis (B) in the H1299 cells that were stably transfected with pcDNA3 or pcDNA3-ABHD5 plasmid. Data are presented as Mean ± SD (n = 3). **p<0.01.
